# The Immunoarchitecture of Human Extraocular Muscles

**DOI:** 10.1167/iovs.64.14.23

**Published:** 2023-11-17

**Authors:** Charlot Philips, Lisanne Terrie, Ewout Muylle, Rita Van Ginderdeuren, Evie Vereecke, Ilse Mombaerts, Lieven Thorrez

**Affiliations:** 1 Tissue Engineering Lab, Department of Development and Regeneration, Catholic University Leuven campus Kulak, Kortrijk, Belgium; 2 Department of Ophthalmology, University Hospitals Leuven, Leuven, Belgium; 3 Jan Palfijn Anatomy Lab, Department of Development and Regeneration, Catholic University Leuven campus Kulak, Kortrijk, Belgium; 4 Department of Neurosciences, Catholic University Leuven, Leuven, Belgium

**Keywords:** extraocular muscle (EOM), T-lymphocyte, B-lymphocyte, macrophage, histopathology

## Abstract

**Purpose:**

The purpose of this study was to describe the immunoarchitecture of normal extraocular muscles (EOMs) in terms of presence, distribution, and organization of various immune cells.

**Methods:**

We performed unilateral orbital exenterations in six fresh human cadavers from elderly patients, followed by dissection of the medial, lateral, superior and inferior rectus, superior and inferior oblique, and superior palpebral levator muscle in their entirety. We further cross sectioned each EOM in an anterior, central, and posterior third. After immunohistochemical staining for CD3, CD8, CD20, CD138, CD68, and podoplanin, quantitative analysis was performed.

**Results:**

We found all EOMs (rectus, oblique, and levator muscles) to harbor both T- and B-lymphocytes, with a B-lymphocyte dominance and an absence of plasma cells. The highest prevalence of immune cells was seen in the muscle bellies, with, on average, 488 ± 63 CD3^+^ T-lymphocytes and 44 ± 110 CD20^+^ B-lymphocytes per mm^2^, and significant differences from the anterior (T-lymphocytes) and posterior (T- and B-lymphocytes) thirds. T- and B-lymphocytes were primarily organized in hotspots in the vicinity of blood vessels. In addition, a small resident population of macrophages scattered throughout the specimens was detected. No lymphatic vessels were found in any of the EOMs.

**Conclusions:**

These findings can serve as a reference dataset in the assessment of EOM biopsies in the diagnostic process of inflammatory orbital and systemic disorders. Moreover, from a regenerative perspective, our results highlight the importance of taking into account the presence of a resident immune cell population when studying the host immune response on transplanted tissues or engineered constructs.

The extraocular muscles (EOMs) are the seven extrinsic muscles of the eye, including four recti and two oblique muscles for ocular motility and one superior palpebral levator muscle for upper eyelid opening. EOMs differ structurally, functionally, biochemically, and immunologically from other skeletal muscles. Diseases of the EOMs include Graves’ orbitopathy, immunoglobulin G4-related disease (IgG4-RD), and idiopathic orbital myositis.^1^^,^^2^ Some muscular diseases are specifically known to preferentially spare (e.g. Duchenne muscular dystrophy and amyotrophic lateral sclerosis) or target EOMs (e.g. myasthenia gravis) in comparison to other skeletal muscles.^3^^–^^6^

The diagnosis of enlarged EOM disease is primarily based on clinical and radiological findings. When these methods are diagnostically indeterminate or atypical, histological analysis of a biopsy of the enlarged EOM is required.^7^^–^^9^ In the histopathological assessment of EOM biopsies in orbital inflammatory disease, a reference normative dataset is currently lacking. Although the histopathology of EOMs in Graves’ orbitopathy,^10^ idiopathic orbital myositis,^11^ and myasthenia gravis^12^ has been studied, little is known about the immunoarchitecture of normal EOMs. Findings from surgical and cadaver EOM biopsies vary widely, from numerous macrophages to total absence of T- and B-lymphocytes. However, the biopsy specimens comprised only small tissue specimens and not all EOMs were studied.^13^^,^^14^

As differences in immunoarchitecture among the recti, oblique, and levator EOMs, and in spatial distribution of each EOM are unknown, we investigated the nature and prevalence of immune cells in normal EOMs, and documented regional differences. This work may serve as a normative data set for diagnosis in EOM biopsies.

## Methods

### Tissue Sampling and Processing

Specimens of the EOMs were obtained from six fresh human cadavers at the Jan Palfijn Anatomy Lab of KU Leuven campus Kulak Kortrijk, within 24 hours after death. There were two males and four female cadavers, aged 65 to 100 years with a mean age of 84.2 years. All subjects had given full and written informed consent to donate their body for medical research. Information regarding the cause of death, comorbidities, and medication use was not available. The study was approved by the Institutional Review Board and Ethical Committee (EC Zorg, NH019-2020-04-02).

**Figure 1.**
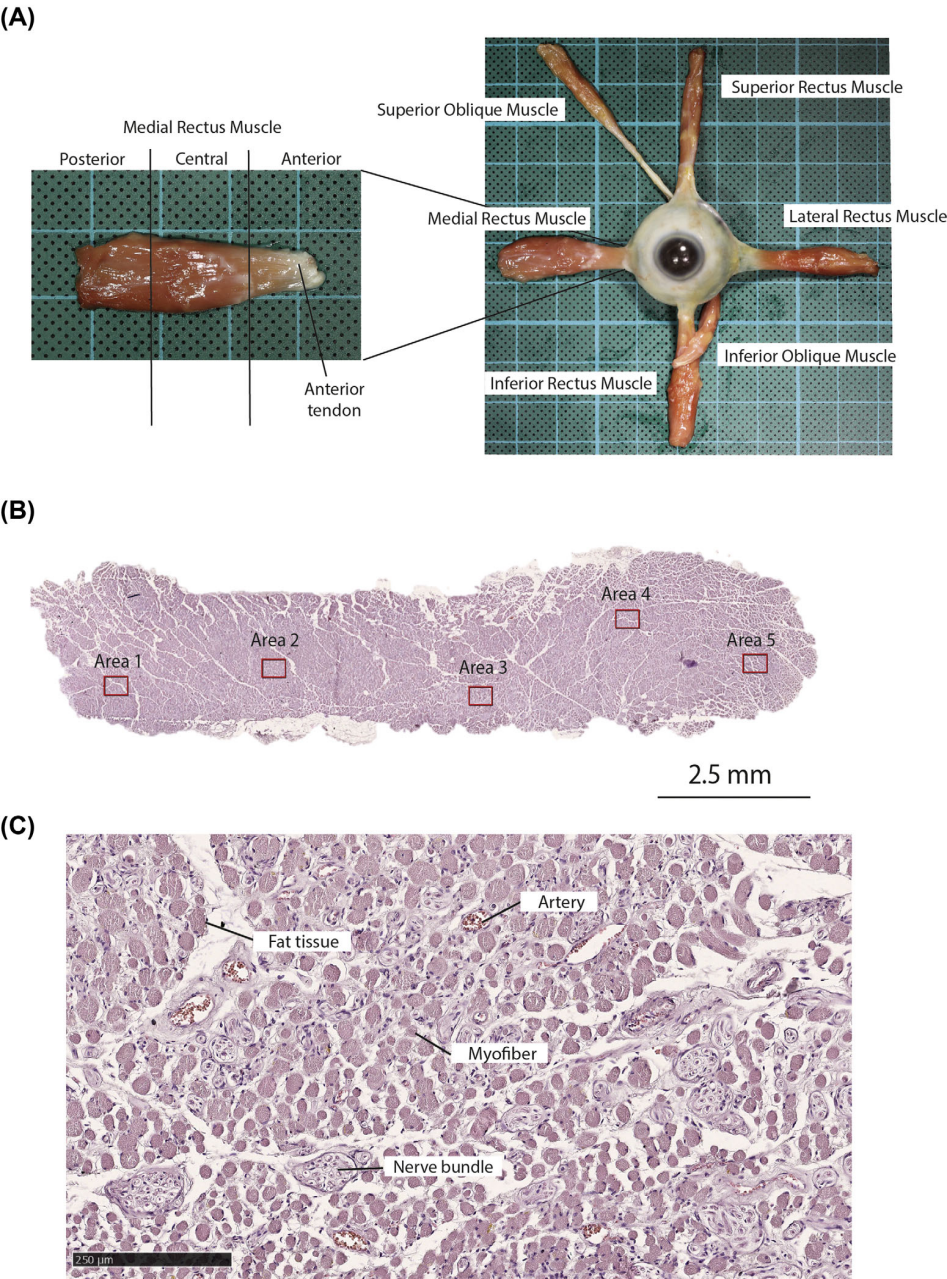
**Obtaining and processing of extraocular muscle tissue.** (**A**) Orbital exenteration specimen of the left side with the exposed EOMs attached to the sclera of the globe. The levator muscle is not shown as this muscle does not attach to the eyeball. The medial rectus muscle is shown here as an example of how all the EOMs were cut along their length in an anterior third (including the anterior tendon), central third, and posterior third.^15^ (**B**) Cross section of a medial rectus muscle stained with hematoxylin and eosin with 5 representative areas of 0.09 mm^2^ for quantitative analysis. (**C**) Higher magnification of a 0.09 mm^2^ area, demonstrating the main structures in EOMs, including myofibers, arteries, nerve bundles, and fat tissue. Scale bar = 250 µm.

The orbital contents including the periosteum were removed in continuity with the eyelids using an orbital exenteration technique. One orbit, selected based on muscle preservation, of each cadaver was exenterated. Via a conjunctival peritomy approach, the rectus and oblique muscles were detached from the globe. With blunt dissection, the muscles were gently separated from the surrounding extraconal and intraconal orbital fat in the direction of the orbital apex (Fig. 1A). The superior palpebral levator muscle was detached from the tarsal plate and separated from Müller's muscle. At the orbital apex, the posterior tendon of the muscles (except for the inferior oblique muscle) could not be preserved because of the common tendinous origin at the annulus of Zinn. The global and orbital layer of the muscles were not marked.

Immediately following sampling, the EOMs were fixed in 4% paraformaldehyde for 24 hours. The muscles were cut at two positions along their anteroposterior length, dividing them into an anterior third (including the anterior tendon), central third (muscle belly), and posterior third (orbital apex). The samples were dehydrated (Leica TP1020), embedded in paraffin (Leica EG1150H), serially cross sectioned (Leica RM2125 RTS) at 5 µm thickness, and mounted on glass slides (superfrost plus, VWR).

### Immunohistochemistry

Cross sections of EOM tissues were immunohistochemically stained to visualize T-lymphocytes, B-lymphocytes, macrophages, and lymphatic vessels. Tissue sections were first rehydrated using the Leica ST5010 Autostainer XL (Leica Biosystems) followed by antigen retrieval with EnVision FLEX Target Retrieval Solution, High pH (Agilent; K800421-2) performed with the PT Link (Agilent). Subsequently, endogenous peroxidase activity was blocked with Peroxidase-Blocking Solution (Agilent; S202386-2). Primary antibodies were incubated for 30 minutes at room temperature and included FLEX Polyclonal Rabbit Anti-Human CD3 (Agilent; IR50361-2), FLEX Monoclonal Mouse Anti-Human CD8, Clone C8/144B (Agilent; IR62361-2), FLEX Monoclonal Mouse Anti-Human CD20cy, Clone L26 (Agilent; IR60461-2), FLEX Monoclonal Mouse Anti-Human CD68, Clone KP1 (Agilent; IR60961-2), FLEX Monoclonal Mouse Anti-Human CD138, Clone MI15 (Agilent; IR64261-2), and FLEX Monoclonal Mouse Anti-Human Podoplanin, Clone D2-40 (Agilent; IR07261-2). Next, EnVision^+^ Dual Link System-HRP Rabbit/Mouse (Agilent; K406189-2) was used as the secondary antibody for 30 minutes at room temperature, after which the peroxidase reaction was visualized with the Liquid DAB^+^ Substrate Chromogen System (Agilent; K346811-2). Finally, counterstaining with hematoxylin was performed using the Leica ST5010 Autostainer XL followed by extensive washing, dehydration, and mounting (CV5030 Glass Coverslipper; Leica Biosystems). Images were acquired with Nanozoomer-SQ (Hamamatsu) and visualized with NDP.view2 software.

### Quantitative Image Analysis

Histological sections were quantitatively analyzed by selecting 5 representative areas of 0.09 mm^2^ in which positive cells were counted (Fig. 1B). For each immunohistochemical staining, there was a total of 630 measurements (6 orbital specimens × 7 EOMs × 3 third parts × 5 areas). In addition to counting absolute numbers of positive cells, the presence of hotspots was also registered. A hotspot was defined as five or more positive cells in close proximity to each other. Both the number of hotspots and the size of the hotspots were used for further analysis.

### Statistical Analysis

All quantitative data were statistically processed with GraphPad Prism version 9.3.1. Normality of the data was assessed with a Shapiro-Wilk test. Differences between groups in cell density and cell distribution were analyzed with a Kruskal-Wallis test followed by Dunn's multiple comparison. For quantitative analysis of hotspots, a two-way Analysis of Variance was performed followed by Tukey's multiple comparison. Average numbers of positive cells per EOM or region are represented as scatter plots with individual values and indication of the median, whereas the size and number of hotspots is represented as histograms. Despite the nonparametric distribution of the values for cell density and cell distribution, it was chosen to present the mean ± standard deviation instead of the median throughout the manuscript for clarity.

## Results

**Figure 2.**
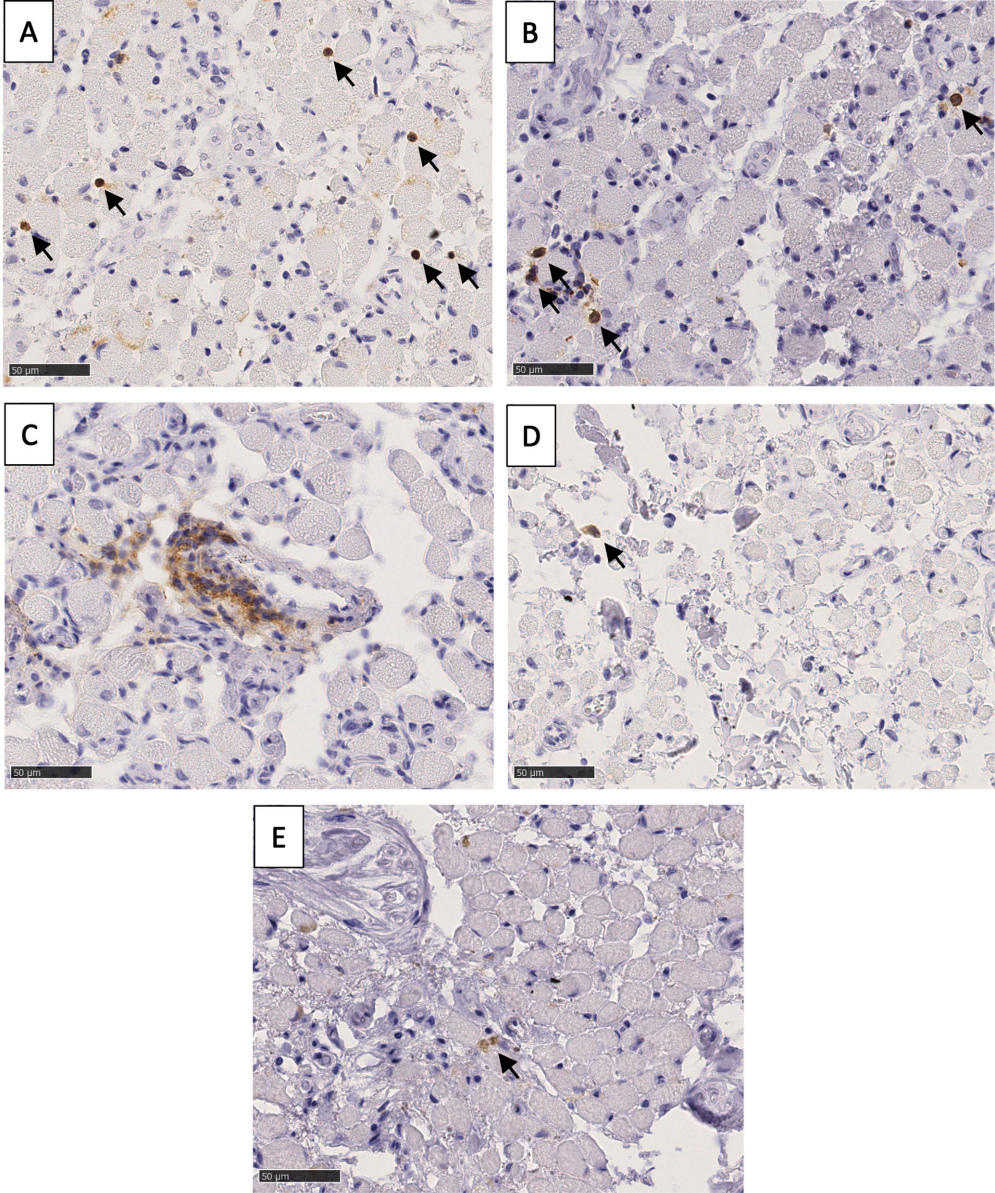
**Immunohistochemical staining set of a medial rectus muscle.** The presence of immune cell markers is shown by a brown DAB (3,3′-diaminobenzidine) staining with a hematoxylin counterstain. (**A**) CD3 staining demonstrating infiltration with T-lymphocytes (*black arrows*). (**B**) CD8 staining showing the presence of a subpopulation of cytotoxic T-lymphocytes (*black arrows*). (**C**) CD20 staining demonstrating infiltration with B-lymphocytes. (**D**) CD138 staining revealing a plasma cell (*black arrow*). (**E**) CD68 staining demonstrating a cluster of irregularly shaped macrophages (*black arrow*). Scale bar = 50 µm.

Through orbital exenteration, we isolated the EOMs of six human donors. Macroscopically, all EOM specimens showed adequate tissue preservation without signs of a pathological process. After tissue fixation, we divided each EOM into an anterior third, central third (muscle belly), and posterior third (orbital apex). The 126 samples (7 EOMs × 3 third parts × 6 donors) were processed for immunohistochemistry. Of each sample, immunohistochemical staining was performed for five markers (CD3, CD8, CD20, CD138, and CD68) which are commonly used to investigate immune cell infiltration (Fig. 2). For T-lymphocytes, both CD3 (present on both cytotoxic as well as T-helper cells) and CD8 (cytotoxic T-cells) were used as markers. Both stainings showed T-lymphocytes as intensely stained, clearly rounded cells (Figs. 2A, 2B), which could be counted individually. For CD20^+^ B-lymphocytes the staining was more diffuse (Fig. 2C) and cells were exclusively found clustered. Therefore, we also quantified the number of hotspots (containing ≥ 5 cells). A CD138^+^ plasma B-cell was recognized as a diffusely stained cell with a clear nucleus (Fig. 2D). Through staining of CD68, macrophages were observed as irregularly shaped cells (Fig. 2E).

**Figure 3.**
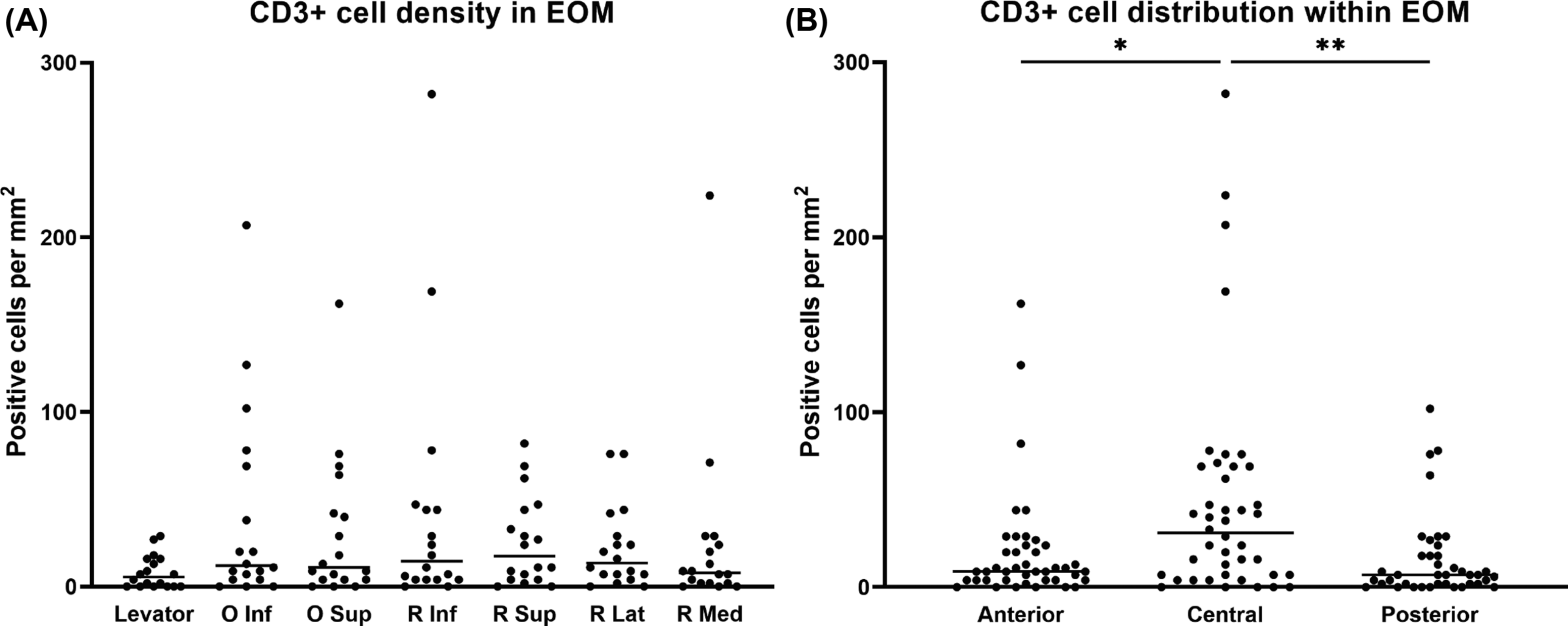
**Presence and distribution of CD3^+^ T-lymphocytes.** (**A**) T-lymphocytes are found in all EOMs without statistically significant differences between groups. Each dot represents the average of five areas counted (*n* = 18: 6 donors × 3 regions). (**B**) A significantly higher number of T-lymphocytes is observed in the central region of the EOM tissue compared to the anterior and posterior regions (*n* = 42: 6 donors × 7 muscles). *P < 0.05, **P < 0.01.

**Figure 4.**
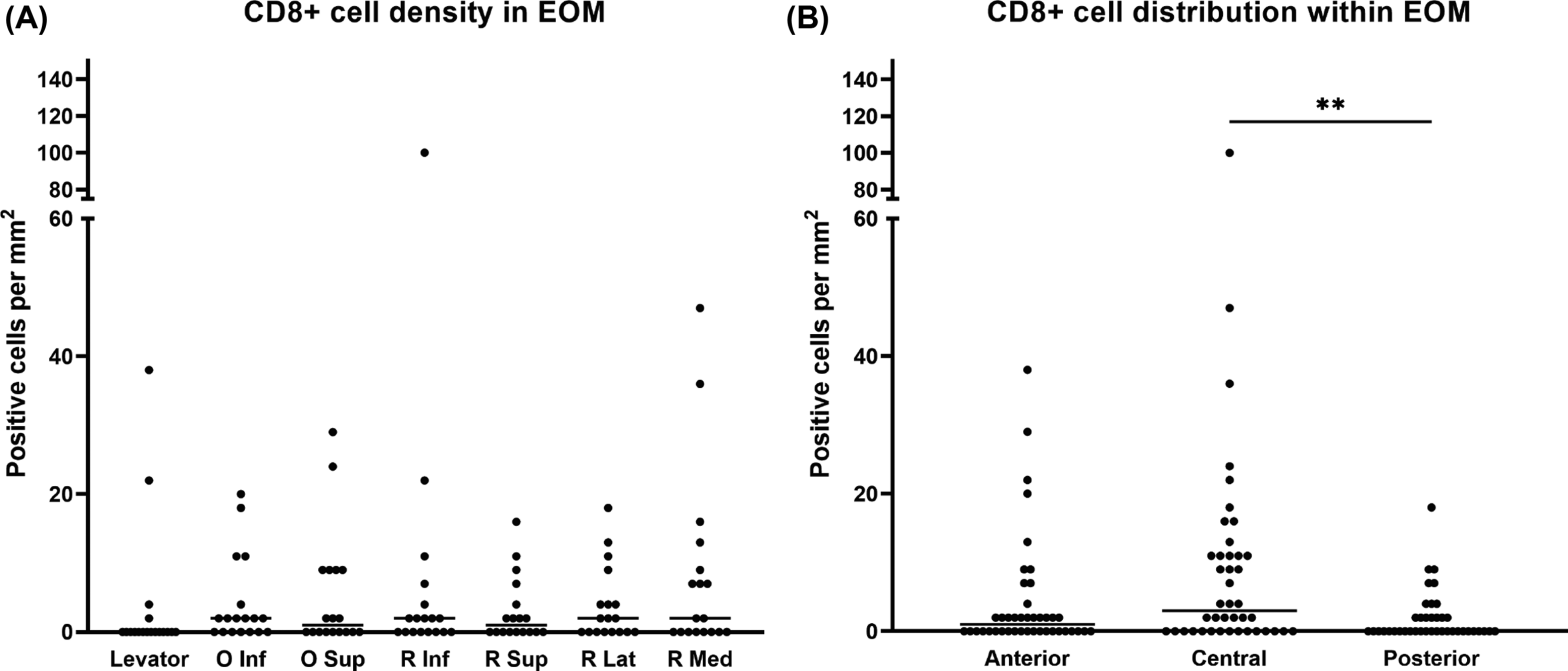
**Presence and distribution of CD8^+^ T-lymphocytes.** (**A**) CD8^+^ T-lymphocytes are present in all EOMs without statistically significant differences between groups. Each dot represents the average of five areas counted (*n* = 18: 6 donors × 3 regions). (**B**) The highest number of CD8^+^ T-lymphocytes is observed in the central region of EOMs, which is significantly different compared to the posterior region (*n* = 42: 6 donors × 7 muscles). **P < 0.01.

T-lymphocytes were present in the EOM tissue of all specimens with mean numbers for CD3^+^ cells ranging from 8 ± 9 to 43 ± 72 cells per mm^2^ and for CD8^+^ T cytotoxic cells from 3 ± 5 to 9 ± 23 cells per mm^2^ (Figs. 3A, 4A). The lowest number of T-lymphocytes was encountered in the superior palpebral levator muscle, but was statistically not different to those in the other muscles. In contrast, the distribution of the cells within the EOMs was significantly different. Increased numbers of CD3^+^ cells were observed in the muscle belly (48 ± 63 cells per mm^2^), which was statistically significant compared to the anterior (20 ± 32 cells per mm^2^, *P* = 0.0452) and posterior (16 ± 24 cells per mm^2^, *P* = 0.0017) region (Fig. 3B). As for the CD8^+^ cytotoxic T-lymphocytes, no significant differences were found between the different EOMs, but there was a difference in cell numbers when comparing the three regions. The muscle belly (10 ± 18 cells per mm^2^) contained significantly more CD8^+^ cytotoxic T-lymphocytes compared to the posterior region (2 ± 4 cells per mm^2^, *P* = 0.0043; Fig. 4B).

**Figure 5.**
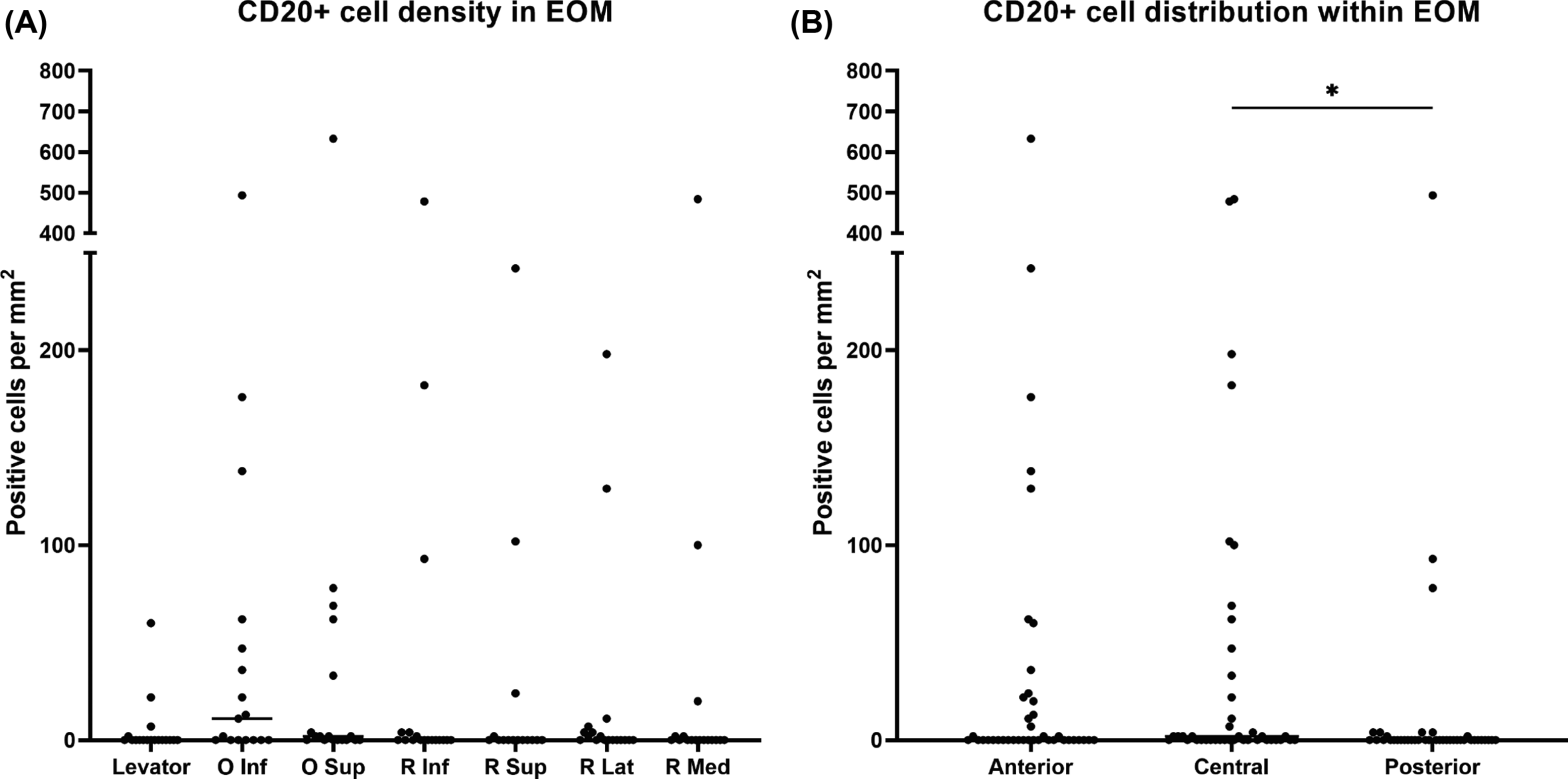
**Presence and distribution of CD20^+^ B-lymphocytes.** (**A**) B-lymphocytes are present in all EOMs without statistically significant differences between the different muscles (*n* = 18: 6 donors × 3 regions). (**B**) An equal number of B-lymphocytes is observed between the anterior and central regions of the EOMs. The posterior had significantly fewer B cells compared to the belly (*n* = 42: 6 donors × 7 muscles). *P < 0.05.

Similar to T-lymphocytes, the presence of B-lymphocytes did not significantly differ between the individual EOMs (Fig. 5A). We observed the lowest absolute number in the superior palpebral levator muscle with a mean of 5 ± 15 cells per mm^2^. For the other EOMs, average numbers ranged from 20 ± 54 to 59 ± 123 cells per mm^2^. The distribution of B-lymphocytes was similar between the anterior (39 ± 110 cells per mm^2^) and central region (44 ± 110 cells per mm^2^), whereas a lower number of cells was found in the posterior region (17 ± 79 cells per mm^2^), which was statistically significant compared to the muscle belly (*P* = 0.0287; Fig. 5B). Plasma cells were virtually absent: 2 single CD138^+^ cells were sampled in the inferior rectus muscle and one in the medial rectus (see Fig. 2D). Because of the scarcity of plasma cells, additional analysis for IgG and IgG4^+^ subtyping was not executed.

**Figure 6.**
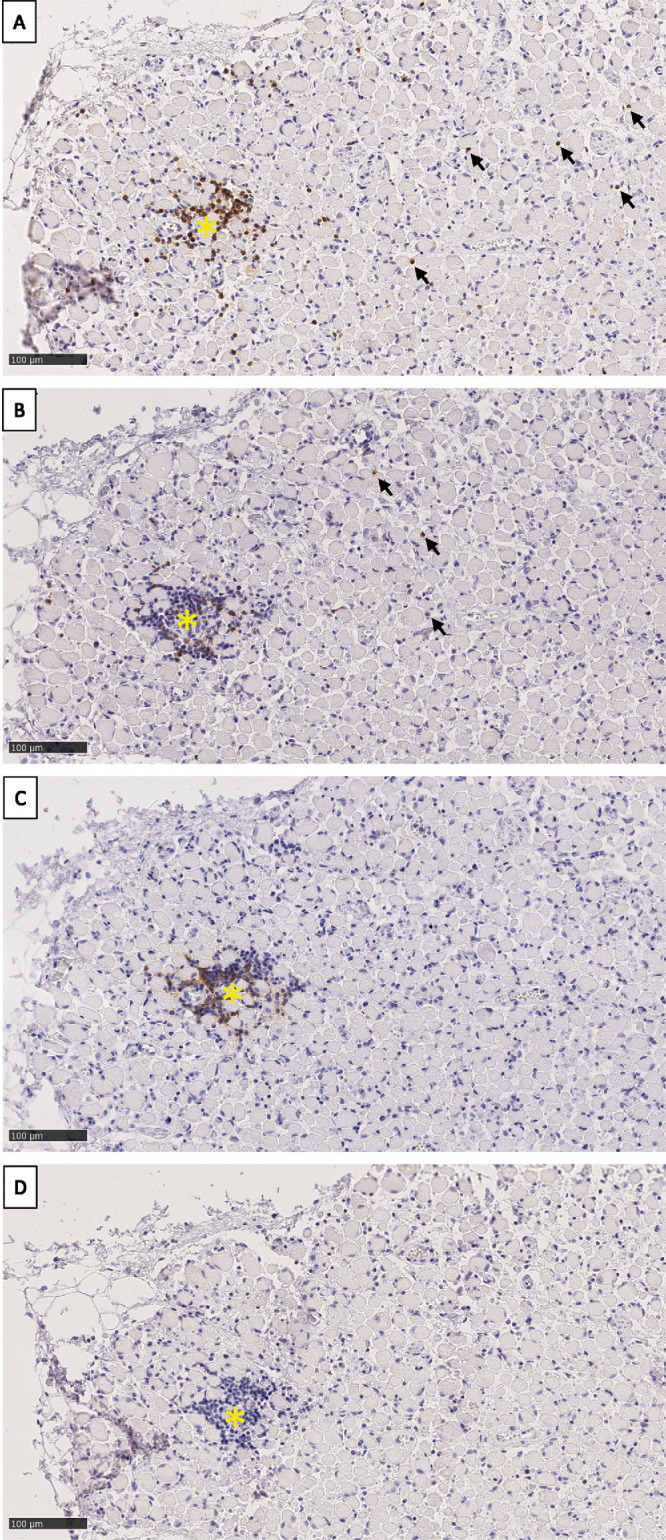
**Hotspot of T- and B-lymphocytes, inferior rectus muscle belly.** (**A**) CD3^+^ cells are present in the hotspot (*yellow asterisk*) and dispersed in the muscle tissue (*black arrows*). (**B**) CD8^+^ cells are mainly found within the hotspot (*yellow asterisk*) with some individual cells present at a distance (*black arrows*). (**C**) CD20^+^ cells are confined to the hotspot (*yellow asterisk*). (**D**) CD138 staining showing the absence of plasma cells in the hotspot (*yellow asterisk*). Scale bar = 100 µm.

In addition to evaluating mean cell numbers per mm^2^, we investigated the spatial distribution of positive cells within the EOM. We observed that most T- and B-lymphocytes were located in hotspots, which were mainly present in the vicinity of a blood vessel (Fig. 6). Scarce single T-lymphocytes were found scattered throughout the specimen, at a distance and separated from the hotspots. In contrast, CD20^+^ B-lymphocytes were almost exclusively found within the hotspots. None of the hotspots contained CD138^+^ plasma cells.

**Figure 7.**
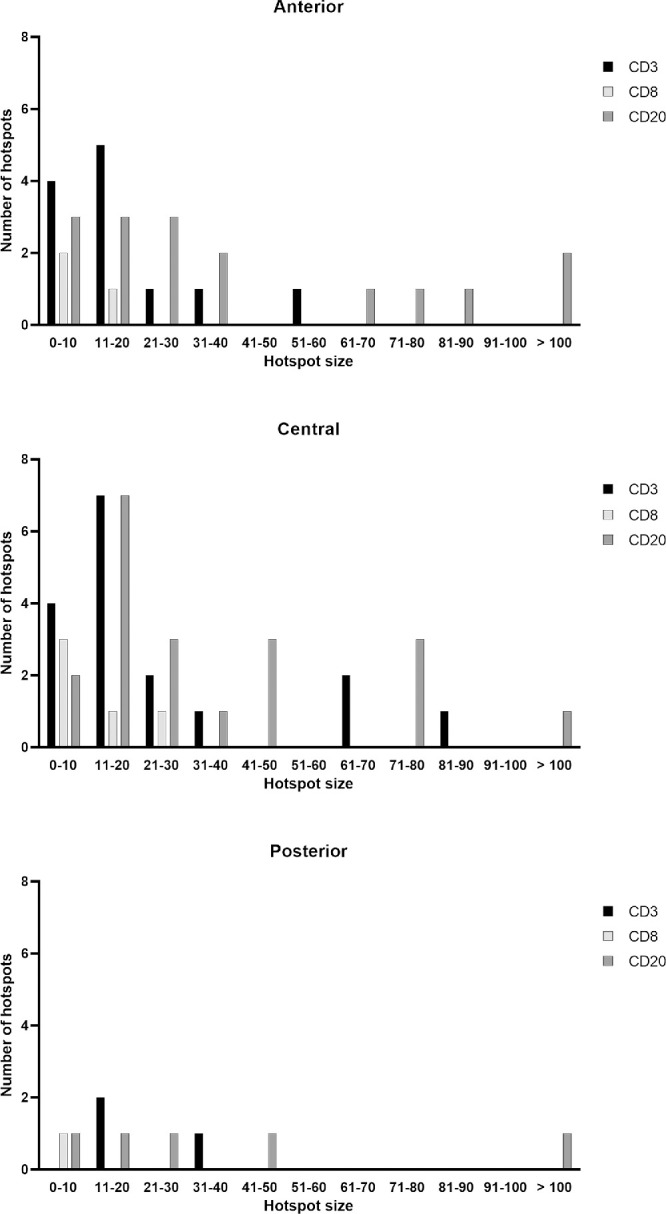
**Number and size of hotspots of T- and B-lymphocytes.** The anterior and central regions of the EOMs contain several hotspots with CD3^+^ or CD20^+^ cells, whereas CD8^+^ hotspots are found to a lesser extent. Hotspots are scarce in the posterior part of EOM tissues.

The size of the hotspots based on the absolute numbers of CD3^+^, CD8^+^, and CD20^+^ cells and the number of hotspots are depicted in Figure 7. Hotspots were significantly increased in the central region compared to the posterior region for both CD3 (*P* = 0.0197) and CD20 (*P* = 0.0273) staining. There were significantly fewer hotspots containing CD8^+^ cells compared to CD3^+^ (*P* = 0.0271) and CD20^+^ (*P* = 0.0013) cells. In addition, the absolute number of CD8^+^ cells within a hotspot was lower than the absolute number of CD3^+^ or CD20^+^ cells. Spread over all 3 regions of the EOM, CD20^+^ cells appeared more frequently in a larger-sized hotspot (40 cells or more) in comparison to CD3^+^ cells.

**Figure 8.**
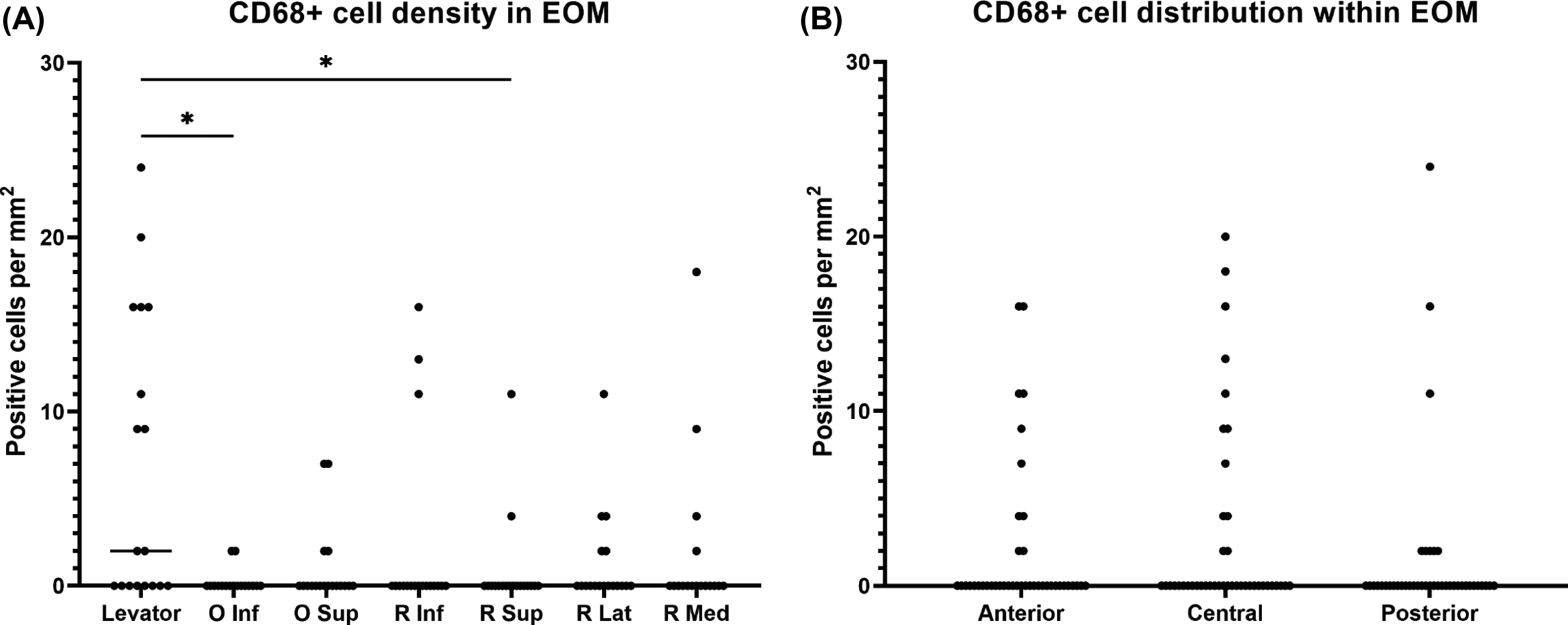
**Distribution of macrophages.** (**A**) Macrophages are seen in all EOM tissues, with the highest absolute number in the superior palpebral levator muscle. The difference is statistically significant when comparing the levator with the inferior oblique or the superior rectus muscle (*n* = 18: 6 donors × 3 regions). (**B**) A slightly higher number of macrophages is observed in the belly of the EOMs, but not significantly compared to the anterior and posterior regions (*n* = 42: 6 donors × 7 muscles). *P < 0.05.

Using CD68 immunostaining, CD68^+^ cells or macrophages were encountered in all EOM tissues (Fig. 8). The absolute numbers were low, with a maximum of 24 cells per mm^2^. The superior palpebral levator muscle accounted for the highest CD68^+^ cell numbers, which was significantly different to the inferior oblique (*P* = 0.0150) and superior rectus (*P* = 0.0179) muscle. The superior palpebral levator muscle had a mean cell number of 7 ± 8 per mm^2^, whereas mean cell numbers for the other EOM ranged from 0 ± 1 to 2 ± 5 cells per mm^2^. No significant differences were seen among the three regions of the EOMs, despite a slightly higher cell count in the belly. Moreover, all macrophages were scattered throughout the muscle tissue and were not organized in hotspots.

**Figure 9.**
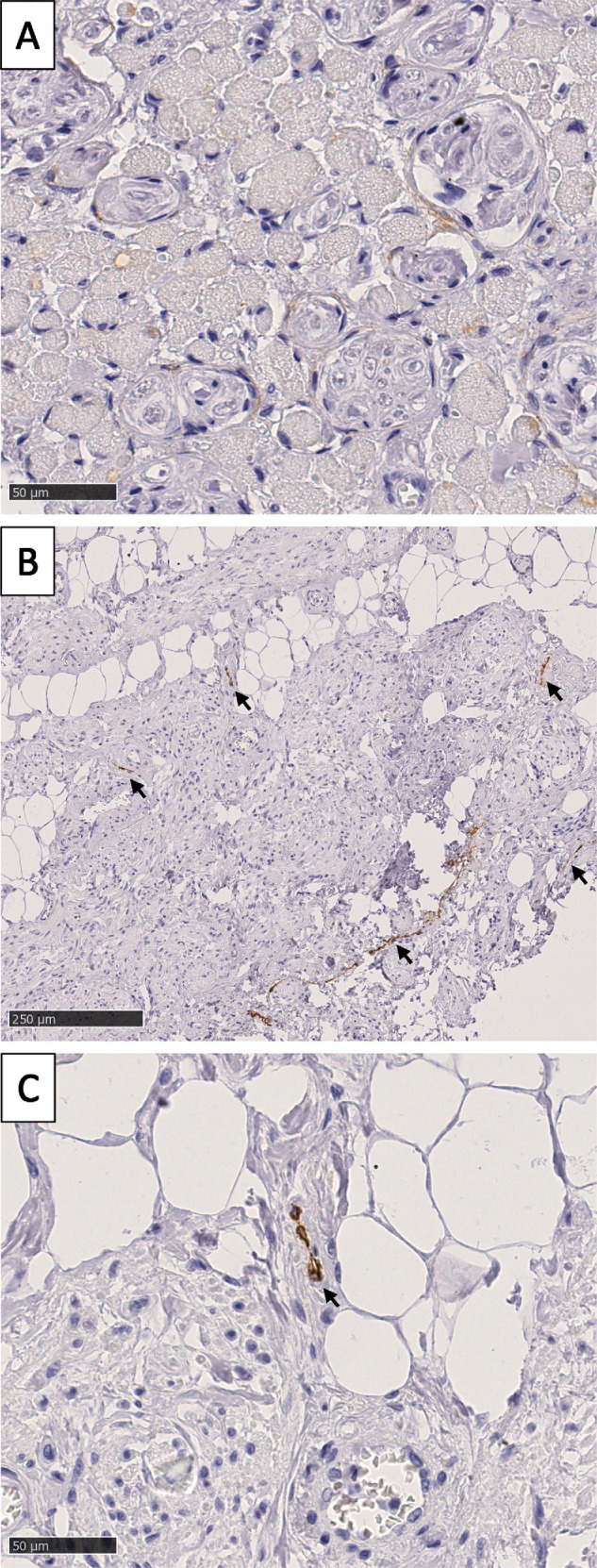
**Lymphatic vessels in the muscle belly and connective tissue of EOM.** (**A**) No lymphatic vessels are seen in the belly of the medial rectus muscle. Scale bar = 50 µm. (**B**) Podoplanin staining showing several lymphatic vessels in the connective tissue adjacent to the anterior part of the superior palpebral levator muscle (*black arrows*). Scale bar = 250 µm. (**C**) Detailed image of a lymphatic vessel demonstrating the collapsed lumen (black arrow). Scale bar = 50 µm.

We stained the EOM specimens with podoplanin as a marker for lymphatic vessels. No lymphatic vessels were found in any specimen (Fig. 9). In the connective tissue adjacent to, but not in, the anterior part of the superior palpebral levator muscle, lymphatic vessels were identified as long, irregular structures with a collapsed lumen, confirming that the staining was carried out properly and hence serving as a control.

## Discussion

With the evolving critical role of EOM biopsy for diagnosis and targeted therapy in orbital inflammatory disease, reference datasets of the inflammatory profile of normal EOMs are needed. This systematic and quantitative immunohistochemistry study of normal EOMs of elderly cadavers shows evidence for a resident mixed lymphocytic, plasma cell-negative infiltrate with a vascular relationship.

Previously reported data on resident immune cells in normal EOMs are scarce. In a study of nine surgical and cadaveric normal EOM specimens, Schmidt et al.^13^ documented the presence of T-lymphocytes in the medial rectus, lateral rectus, inferior rectus, and superior oblique muscles, mainly localized in the belly region and organized around the blood vessels. The authors did not observe B-lymphocytes in the specimens. In the present study, we found the presence of both T-lymphocytes and B-lymphocytes, and in a similar distribution pattern primarily in the vicinity of blood vessels. This finding confirms the role of the vascular system in the mediation of immunocompetent cells in the absence of lymphatic vessels. With regard to subtyping of T-lymphocytes, we found a small fraction of CD8^+^ suppressor/cytotoxic cells. Assuming that the remaining CD3^+^ cells are of the helper (CD4^+^) subtype, this corresponds to a CD4:CD8 ratio higher than 1 and can be considered as normal. However, in Schmidt et al.’s study, a CD4:CD8 ratio of 0.3 to 0.5 was reported.^13^ This difference may reflect the presence of underlying systemic immunodeficiency in the examined donors of the latter study.

Macrophages were present and equally distributed among the EOMs in our study. They play a crucial role in inflammation, repair, and regeneration of tissues, and their presence is well-known in skeletal muscles, approximating one macrophage per five myofibers.^16^^–^^19^ In the quantitative EOM study by Schmidt et al.,^13^ macrophages were abundant, with the medial and inferior recti muscles containing twice as many as the lateral rectus and superior oblique muscles. Furthermore, the number of macrophages greatly exceeded those of T-lymphocytes in their study. Together with the predominance of CD8^+^ cytotoxic T-lymphocytes over CD4^+^ helper T-lymphocytes, these findings may be suggestive of an ongoing chronic inflammatory process. In another immunohistological study of five incisional biopsy specimens of normal EOMs, Tallstedt and Norberg reported scarce macrophages, without T- and B-lymphocytes.^14^ The considerable differences with our study may be attributed to sampling error, as the EOMs were not examined in their entirety in the Schmidt et al.’s and the Tallstedt and Norberg's studies.

In diagnostic EOM biopsies, in-depth pathological analysis and clinico-radiological correlation is essential. The literature reports a few studies on EOM biopsies of patients with idiopathic orbital myositis, Graves’ orbitopathy, and IgG4-RD. Ben Artsi et al*.*^11^ studied nine muscle biopsy specimens of patients with idiopathic orbital myositis. They found in all patients a mixed, predominant B-cell infiltrate, similar to the hotspots observed in our normal EOMs. The authors concluded that the presence of plasma cells and histiocytes, and, less common, eosinophils, neutrophils, and giant cells, along with mild fibrosis, correlated with the diagnosis of idiopathic orbital myositis. In EOMs affected by Graves’ disease, T-cells are significantly present in the active stage, with fibroblasts and fibrosis appearing in early and late disease.^10^^,^^20^

In orbital IgG4-RD, tissue IgG4^+^ plasma cells are an important marker, with the diagnosis based on the presence of a dense lympho-plasmocytic infiltrate and a minimum of 50 IgG4^+^ plasma cells per high power field.^21^ Our EOM specimens were all devoid of plasma cells, hence, the finding of these cells may indicate a diagnosis of IgG4-RD, provided the other histopathological criteria are met.

Orbital diseases preferentially affect specific EOMs. In Graves’ orbitopathy, the relative frequency of involvement of each EOM is inferior rectus > medial rectus > superior rectus/levator > lateral rectus > obliques, whereas idiopathic orbital myositis and lymphoproliferative disease have a predeliction for the horizontal recti muscles.^2^ In this study, we did not find a significant difference in immunoarchitecture among the EOMs. It may, therefore, be concluded that resident immune cells unlikely play a role in the pattern of affected EOMs in these conditions.

Understanding the immunoarchitecture of EOMs is not only useful in pathology, but is also of importance for future strategies in regenerative medicine. Tissue engineering approaches are emerging to replace or repair damaged muscles.^22^^,^^23^ Such approaches include the development of bio-artificial muscle based on fibrin hydrogels or the use of acellular matrices obtained through decellularization.^24^ Little is known, however, about the interaction of the host immune system with engineered constructs in terms of remodeling and reconstruction. In this regard, knowledge on the resident population of immunocompetent cells is a critical first step toward EOM engineering.

There are several limitations of the study inherent to the design. The donors were older than 65 years. As aging changes the histological appearance of EOMs, it can be questioned if our observations can be extrapolated to younger patients.^25^ Further, diseases and drugs can alter immunity and hence the immunocompetent cellular spectrum in the muscles. Inherent to the donation protocol, information regarding cause of death, underlying diseases, and use of drugs of the donors remained unknown to us. However, macroscopically, the size and shape of all muscles appeared normal without muscle or tendon enlargement, or reduced elasticity. In addition, all seven EOMs of all six orbits immunohistologically displayed similar patterns, supporting the likelihood of representative, normal EOMs studied. Last, we did not describe morphological features of the myofibers and the presence of fibrosis, sclerosis, or fatty infiltration, which are topics of future studies.

In conclusion, this study provides new insights into the inflammatory profile of normal human EOMs of the elderly. The recti, oblique, and levator muscles contained perivascular mixed inflammatory aggregates with a B-lymphocyte predominance, particularly localized in the belly region of the muscle, and devoid of plasma cells. From this study, the single finding of a mixed B-cell dominant lymphocytic infiltrate in an EOM biopsy specimen cannot be regarded as a pathological finding. In contrast, a cellular composition of plasma cells, eosinophils, neutrophils, giant cells, and fibroblasts is indicative of an orbital inflammatory condition. In the light of our findings, analysis of EOM biopsies can be better understood to reach a histopathological diagnosis.
